# Limitations of MIC as sole metric of pharmacodynamic response across the range of antimicrobial susceptibilities within a single bacterial species

**DOI:** 10.1038/srep37907

**Published:** 2016-12-01

**Authors:** Xuesong Wen, Ronette Gehring, Andrea Stallbaumer, Jim E. Riviere, Victoriya V. Volkova

**Affiliations:** 1 Department of Anatomy and Physiology, Institute of Computational Comparative Medicine, College of Veterinary Medicine, Kansas State University, Manhattan, KS 66506, USA; 2 Department of Diagnostic Medicine/Pathobiology, Institute of Computational Comparative Medicine, College of Veterinary Medicine, Kansas State University, Manhattan, KS 66506, USA

## Abstract

The minimum inhibitory concentration (MIC) of an antimicrobial drug for a bacterial pathogen is used as a measure of the bacterial susceptibility to the drug. However, relationships between the antimicrobial concentration, bacterial susceptibility, and the pharmacodynamic (PD) inhibitory effect on the bacterial population are more complex. The relationships can be captured by multi-parameter models such as the *E*_max_ model. In this study, time-kill experiments were conducted with a zoonotic pathogen *Pasteurella multocida* and the fluoroquinolone enrofloxacin. *Pasteurella multocida* isolates with enrofloxacin MIC of 0.01 μg/mL, 1.5 μg/mL, and 2.0 μg/mL were used. An additive inhibitory *E*_max_ model was fitted to the data on bacterial population growth inhibition at different enrofloxacin concentrations. The values of PD parameters such as maximal growth inhibition, concentration achieving a half of the maximal inhibition, and Hill coefficient that captures steepness of the relationships between the concentration and effect, varied between the isolate with low MIC and less susceptible isolates. While enrofloxacin PD against the isolate with low MIC exhibited the expected concentration-dependent characteristics, the PD against the less susceptible isolates demonstrated time-dependent characteristics. The results demonstrate that bacterial antimicrobial susceptibility may need to be described by a combination of parameters rather than a single parameter of the MIC.

Effective antimicrobial treatment regimens for bacterial infections are critical for achieving the therapeutic outcomes and minimizing development of bacterial antimicrobial resistance. Designing the regimens requires knowledge of the pharmacokinetics and pharmacodynamics of the antimicrobial drug in the host treated. The pharmacokinetic models project the drug concentrations over time in the bodily compartments where the infection is treated. The pharmacodynamics (PD) is how those concentrations affect the pathogen population. The antimicrobial’s minimum inhibitory concentration (MIC) for the pathogen is used as a standardized measure of the bacterial susceptibility to the drug (the minimum concentration that inhibits overnight visible growth of the bacterial isolate’s population culture when exposed to the drug at a standardized starting density). Then, the time the drug concentration in the infected organ remains above the MIC is considered for projecting the pharmacodynamic effect against the pathogen population, and choose the dosing interval, for an antimicrobial which effect is expected to be time-dependent^1^,^2^. For an antimicrobial which effect against the pathogen population is expected to be concentration-dependent, the ratio of the peak drug concentration in the organ to the MIC and the area under the curve over the time when the concentration remains above MIC are considered^1^,^2^. Consequently, the dosing regimens for antimicrobial drugs that show a concentration-dependent PD for the pathogen tend to constitute higher doses administered less frequently, whereas those exhibiting a time-dependent relationship tend to constitute smaller doses administered more frequently.

However, relationships between the antimicrobial concentration, bacterial susceptibility, and the PD-effect on the bacterial population are known to be more complex than what can be captured by the MIC parameter alone. Multi-parameter mathematical models have been developed to capture the relationships, such as the maximum effect (*E*_max_) model based on the Hill function^3^,^4^. These models linked with those of the *in vivo* drug pharmacokinetics may better capture the microbiological efficacy of the dynamic antimicrobial exposure^5^,^6^. Still, once the *E*_max_ model’s form is chosen and its parameters’ values estimated for activity of the antimicrobial against the pathogen, the model is applied to different pathogen isolates under the assumption that the MIC is the only parameter that changes. In other words, within a given bacterial species, the values of the other PD parameters are assumed to be constant across the isolates with various MIC of the drug. Based on our review of experimental literature^7^, we hypothesized that this is not the case; the other PD parameters’ values may change between pathogen isolates for which the drug’s MIC differ. This can have significant clinical implications for design of antimicrobial treatment regimens, as then not only the MIC but also other PD parameters need to be considered when choosing the dose and dosing interval. Recent experimental evidence confirms this hypothesis for commensal *Escherichia coli* and a time-dependent antimicrobial tetracycline^8^. For example, larger values of the PD parameter Hill coefficient are observed for *E. coli* isolates with higher tetracycline MICs, compared to the isolates with lower MICs^8^. The value captures the steepness in relationships between an increase in the antimicrobial concentration and an increase in the PD-effect on growth of the exposed bacterial population.

The objective of this study was to test the hypothesis that for pathogenic bacteria’s isolates with different MIC of an antimicrobial drug, the values of the other PD parameters for the drug also differ. We experimented with *Pasteurella multocida*, which is both an important pathogen of animals, in particular cattle^9^,^10^, and a zoonotic bacteria with a wide host range and commonly present in infected animal wound bites in humans^11^,^12^,^13^. The antimicrobial was the fluoroquinolone enrofloxacin, which is thought to exhibit the concentration-dependent activity against this pathogen^14^.

## Results

### PD model form

**Figure 1:**
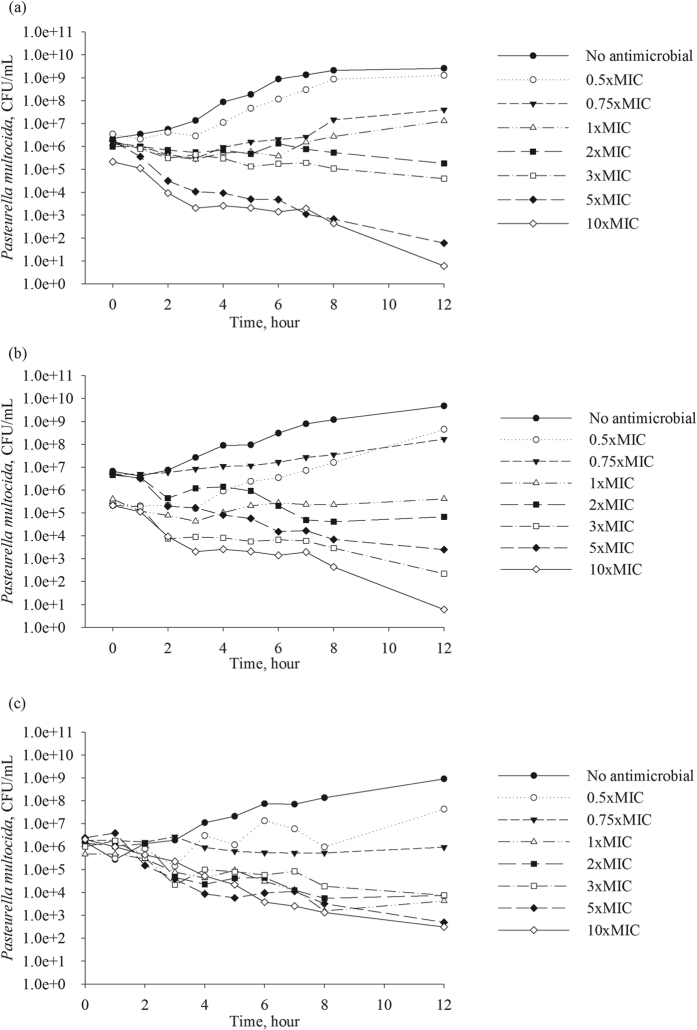
Data from the time-kill experiments for three *Pasteurella multocida* isolates: (**a**) isolate with enrofloxacin MIC = 0.01 μg/mL; (**b**) isolate with enrofloxacin MIC = 1.5 μg/mL; and (**c**) isolate with enrofloxacin MIC = 2.0 μg/mL. Each point is the geometric mean of the bacterial densities in two independent experiments. CFU – colony forming units.

The data from the time-kill experiments are plotted in Fig. 1. Several formulations of the *E*_max_ model were compared for the fit to capture the relationships between the antimicrobial concentration and the rate of growth/decline of the exposed bacterial population. The best fit was observed for all three isolates for an additive inhibitory-effect *E*_max_ model with a sigmoid form for the relationships between an increase in the drug concentration and an increase in the inhibitory effect on the bacterial population growth. The *E*_max_ model is given in equation (1):


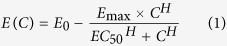


Where:

1. *C* - antimicrobial concentration.
2. *E*(*C*) - bacterial population growth rate when exposed to concentration *C*.
3. *E*_0_ - bacterial population growth rate in the absence of antimicrobial exposure.
4. *EC*_50_ - concentration of the antimicrobial that achieves a 50% of the maximal inhibition of the bacterial population growth rate.
5. *E*_max_ - maximal inhibition of the bacterial population growth rate.
6. *H* - Hill coefficient.

### Parameter values of the PD model

**Figure 2:**
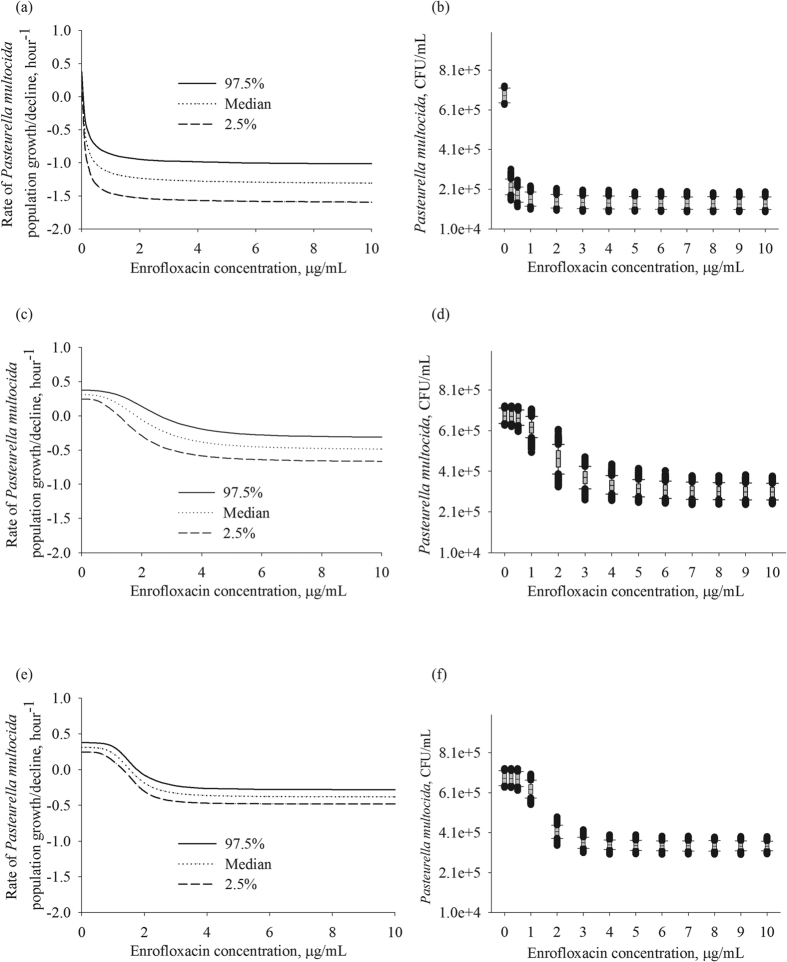
The simulated distributions of the rate of bacterial population growth/decline for three *Pasteurella multocida* isolates: (**a**) isolate with enrofloxacin MIC = 0.01 μg/mL; (**c**) isolate with enrofloxacin MIC = 1.5 μg/mL; and (**e**) isolate with enrofloxacin MIC = 2.0 μg/mL. The resulting bacterial density after 1-hour exposure to different enrofloxacin concentrations, with the starting density 5 × 10^5^ CFU/mL, for three *Pasteurella multocida* isolates: (**b**) isolate with enrofloxacin MIC = 0.01 μg/mL; (**d**) isolate with enrofloxacin MIC = 1.5 μg/mL; and (**f**) isolate with enrofloxacin MIC = 2.0 μg/mL. In each panel or box-plot, the outputs of 1,000 simulations for each enrofloxacin concentration of the best-fit pharmacodynamic model for the isolate are summarized. CFU – colony forming units.

**Figure 3:**
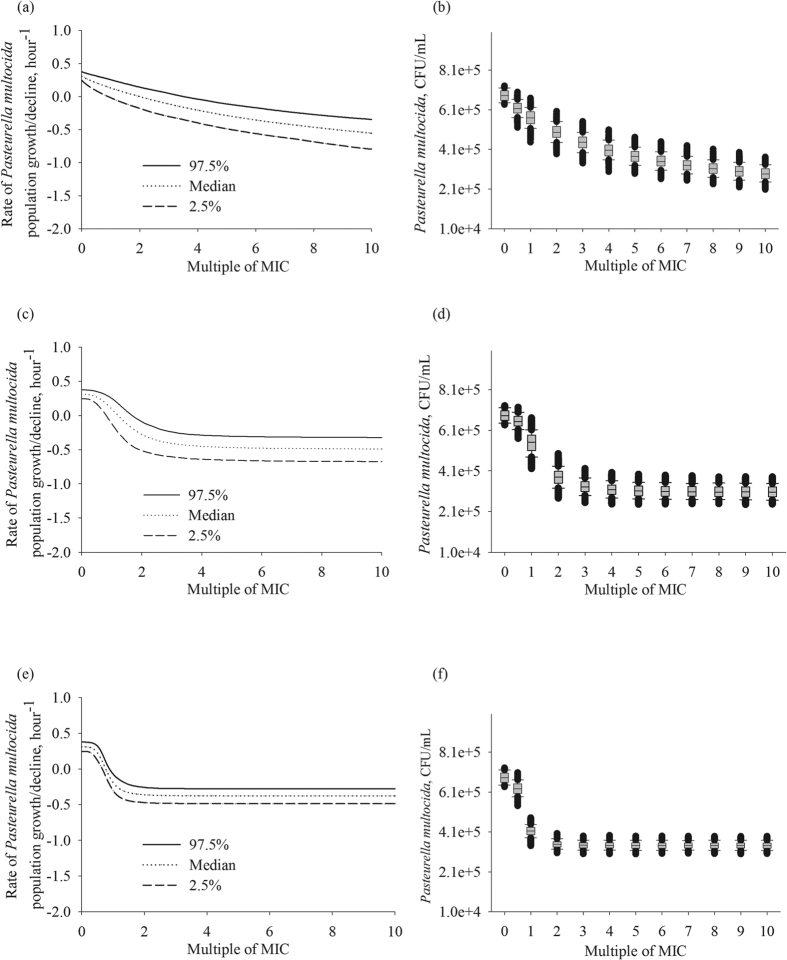
The simulated distributions of the rate of bacterial population growth/decline for three *Pasteurella multocida* isolates: (**a**) isolate with enrofloxacin MIC = 0.01 μg/mL; (**c**) isolate with enrofloxacin MIC = 1.5 μg/mL; and (**e**) isolate with enrofloxacin MIC = 2.0 μg/mL. The resulting bacterial density after 1-hour exposure to different enrofloxacin concentrations standardized as multiples of the isolate’s MIC, with the starting density 5 × 10^5^ CFU/mL, for three *Pasteurella multocida* isolates: (**b**) isolate with enrofloxacin MIC = 0.01 μg/mL; (**d**) isolate with enrofloxacin MIC = 1.5 μg/mL; and (**f**) isolate with enrofloxacin MIC = 2.0 μg/mL. In each panel or box-plot, the outputs of 1,000 simulations for each enrofloxacin concentration of the best-fit pharmacodynamic model for the isolate are summarized. CFU – colony forming units.

The population growth rate in the absence of antimicrobial exposure, *E*_0_, was similar among the three *P. multocida* isolates, averaging at 0.3 per hour (Table 1). Hence, the growth rate appeared to be independent of the MIC. The values of the other PD parameters were statistically significantly different for the isolate with enrofloxacin MIC of 0.01 μg/mL compared to the isolates with the MIC of 1.5 and 2 μg/mL (based on the non-overlapping 95% CI for the parameter estimates, Table 1). The highest maximal inhibition of the *P. multocida* population growth rate of 1.64 was observed for the isolate with enrofloxacin MIC of 0.01 μg/mL. Furthermore, the *EC*_50_ for this isolate was 0.11 μg/mL, which was 10 times higher than its MIC. The inhibition of the bacterial growth steeply increased with relatively small increases in drug concentration (Fig. 2a,b), and continued to increase at the concentrations of high MIC multiples (Fig. 3a,b). This was despite the Hill coefficient’s value close to 1, which otherwise would indicate a low steepness in the relationships between an increase in drug concentration and the bacterial growth inhibition. In contrast, the maximal inhibition of the bacterial population growth rate was 0.81 and 0.69 for the isolates with enrofloxacin MIC of 1.5 and 2 μg/mL, respectively. The *EC*_50_ values were close to the MIC, 2.13 and 1.60 μg/mL, respectively. The inhibitory effect was less sensitive, compared to the more susceptible strain, to relatively small increases in drug concentration (Fig. 2c,d and e,f vs. a,b). This was despite relatively high values of the Hill coefficient, 3.05 and 4.37, respectively. Increases in drug concentration above 3 to 5 multiples of the MIC of the isolates had no further inhibitory effect on the bacterial population growth (Fig. 3c,d and e,f).

The pharmacodynamics of enrofloxacin is considered to be concentration-dependent, with the inhibitory effect on the bacterial population growth sensitive to relatively small increases in drug concentration, and an increase in the inhibitory effect continuing at drug concentrations above low MIC multiples. This indeed was observed for the isolate with enrofloxacin MIC of 0.01 μg/mL, as indicated by the time-kill data (Fig. 1a), the high maximal growth inhibition of 1.64, and the *EC*_50_ value being several folds higher than the MIC (Table 1). The panels Fig. 2a,b visualize that the inhibitory effect on the isolate’s population growth rapidly increased with an increase in the antimicrobial concentration. The panels Fig. 3a,b demonstrate that the increase continued at high MIC multiples. These clearly confirmed the concentration-dependent PD dynamics. In contrast, the time-kill data for *P. multocida* isolates with enrofloxacin MIC of 1.5 and 2 μg/mL (Fig. 1b,c), the low maximal growth inhibition of 0.81 and 0.69, respectively, and the *EC*_50_ values close to the MIC values were suggestive of time-dependent PD. The inhibitory effect on the bacterial population growth was less sensitive to small increases in drug concentration (Fig. 2c,d and e,f) and only increased until the concentrations of low multiples of the MIC, after which the relationships exhibit a saturation with no further increase in the growth inhibition at higher drug concentrations (Fig. 3c,d and e,f).

Also, interestingly, the simulations demonstrated that the possible inhibitory PD effect on bacterial growth and the resulting bacterial density trajectories were more variable for the isolate with enrofloxacin MIC of 0.01 μg/mL, compared to the isolates with higher MIC values (Fig. 2a,b vs. c,d and e,f; Fig. 3a,b vs. c,d and e,f). This was because for the former the Hill coefficient estimate was close to 1 and the maximal inhibition of the growth, *E*_max_, was a relatively high value (Table 1). A random variability in individual bacterial populations could result in a Hill coefficient value above or below 1, and relatively larger deviations in the maximal inhibition. The trajectories of the bacterial populations of the isolates with higher MIC under the antimicrobial exposure appeared more predictable (Figs 2c,e; Fig. 3c,e), likely because of the relatively high Hill coefficient values.

## Discussion

In this study, the maximal inhibition of *P. multocida* population growth (*E*_max_) was statistically significantly higher for the isolate with the lowest enrofloxacin MIC compared to the isolates with higher MIC values. Also for the former, the concentration at which 50% of *E*_max_ was achieved, *EC*_50_, was significantly greater relative to the MIC (Table 1). The inhibition of the population growth of the more susceptible isolate was more sensitive to smaller changes in the antimicrobial concentration, and the effect continued to respond to an increase in the concentrations above several multiples of the MIC (Figs 2a,b and 3a,b). This corresponded to the expected concentration-dependent PD dynamics of enrofloxacin, a fluoroquinolone antimicrobial. In contrast, the inhibition of the population growth for the isolates with higher MIC was less sensitive to increases in the antimicrobial concentration, and the effect plateaued at the concentrations of low MIC multiples (Fig. 2c,d and e,f; Fig. 3c,d and e,f). Thus, the PD dynamics were more in line with time-dependent characteristics. These observations may seem counterintuitive given the estimated Hill coefficient value of 1 for the isolate with the lowest MIC, compared the large Hill coefficient values, 3 to 4, for less susceptible isolates. However, the steepness of the relationships between an increase in the antimicrobial concentration and the inhibitory effect on the pathogen population – the slope of the concentration-effect curve - is dependent on the combination of the PD parameters. In particular, the high Hill coefficient values for the isolates with higher enrofloxacin MIC did not offset the impacts of the low *EC*_50_ and *E*_max_ values (Table 1).

The direction of change in the PD-parameter Hill coefficient among bacterial isolates with different MIC (determined under the standard conditions) of the fluoroquinolone enrofloxacin for zoonotic pathogen *P. multocida* in this study was similar to that reported for commensal *E. coli* for tetracycline^8^. The magnitude of the change was also remarkably similar between the two bacteria-antimicrobial systems. A three to four fold increase in the Hill coefficient value was observed for *P. multocida* isolates with enrofloxacin MIC of 1.5 or 2 μg/mL, compared to the isolate with the MIC of 0.01 μg/mL (Table 1). Based on the data presented in Ahmad *et al*.^8^, the average value of Hill-coefficient for *E. coli* isolates with tetracycline MIC above 2 μg/mL was four times that for the isolates with tetracycline MIC below 0.5 μg/mL. This consistency is notable, given that enrofloxacin exhibits the concentration-dependent PD dynamics against highly susceptible bacterial isolates as described by the MIC^14^ (including in this study for the isolate with the MIC of 0.01 μg/mL). In contrast, tetracycline exhibits the time-dependent PD dynamics irrespectively of the bacterial susceptibility as described by the MIC^8^. Moreover, the fluoroquinolone enrofloxacin is bactericidal but tetracycline is bacteriostatic at the concentrations achievable in the body^15^.

On the other hand, while the value of the PD-parameter *EC*_50_ increased in line with the increase in MIC of the isolates in both these bacteria-antimicrobial systems, the relationships between the *EC*_50_ and MIC for a given isolate differed. For activity of tetracycline against *E. coli*, the *EC*_50_ was below the MIC for majority of the isolates with either a relatively low or high MIC^8^, as could be expected for an antimicrobial with the primarily time-dependent PD. For activity of enrofloxacin against *P. multocida*, the *EC*_50_ was several folds higher than the MIC for the isolate with the lowest MIC studied, 0.01 μg/mL, as could be expected for the concentration-dependent PD. For the isolates with enrofloxacin MIC of 1.5 or 2 μg/mL, the *EC*_50_ was close to the MIC (Table 1), highlighting the change towards more time-dependent characteristics. The maximal inhibition of the bacterial population growth achieved at high antimicrobial concentrations, *E*_max_, was approximately twice less for the *P. multocida* isolates with enrofloxacin MIC of 1.5 or 2 μg/mL, compared to the isolate with the MIC of 0.01 μg/mL in this study (Table 1). This parameter was not reported by Ahmad *et al*.^8^, and therefore could not be compared.

Based on the experimental results of this study and those in Ahmad *et al*.^8^, and also earlier theoretical considerations^16^, a question could be raised about how to define the susceptibility of a bacterial isolate to an antimicrobial. The results jointly demonstrate that susceptibility cannot be simply described by the antimicrobial’s MIC. The effects of the dynamic antimicrobial exposure *in vivo* on the pathogen population also will depend on the responsiveness of the isolate to a change in the drug concentration. The steepness (which depends on the combination of the PD parameters) of the relationships between an increase in the drug concentration and the inhibitory effect on the bacterial population is a feature of the bacterial susceptibility. Thus, the entire combination of PD parameters that reflect the isolate’s susceptibility, not only the MIC, can be taken into account when designing the treatment regimens. This can have significant clinical implications, pertaining to the antimicrobial’s dose and dosing interval needed to address pathogen’s isolates with different susceptibilities. Simple “rules of thumb” may not apply and the complete PD profile for pathogen isolates of different susceptibilities should be considered along with the antimicrobial drug’s pharmacokinetics and toxicological considerations in the host to design effective treatment regimens. The data also suggest that should antimicrobial resistance occur in the pathogen under treatment, a different dosage regimen may be required; this is not currently being considered in clinical settings.

## Methods

### Bacterial isolates and antimicrobial agent

Three isolates of *P. multocida* from respiratory organs of cattle were obtained from the Kansas State Veterinary Diagnostic Laboratory. Enrofloxacin (Sigma-Aldrich, St. Louis, MO, USA) stock solutions (1 mg/mL) were prepared in methanol.

### MIC estimation

Susceptibility of each isolate to enrofloxacin was evaluated by measuring the MIC by the broth microdilution method performed in accordance with the Clinical and Laboratory Standards Institute’s recommendations^17^, and by the E-test^®^ performed in accordance with the manufacturer’s recommendations (bioMérieux, Marcy-l'Étoile, France). An isolate with enrofloxacin MIC = 0.01 μg/mL, another with MIC = 1.5 μg/mL, and a third isolate with MIC = 2 μg/mL were included in the time-kill experiments.

### Time-kill experiments

The time-kill protocol was an adaptation of that previously described^18^. Briefly, bacteria were grown aerobically in 20 mL of brain heart infusion broth (BHI, Remel^TM^, Lenexa, KS, USA) at 37 °C with shaking (200 rpm) overnight. The overnight cultures were diluted 1:200 into fresh BHI and incubated at 37 °C for 30 minutes, and then diluted 1:20 into flasks containing fresh BHI with different concentrations of enrofloxacin for the time-kill experiments. The starting bacterial densities in the experiments were 10^5^ to 10^6^ colony forming units (CFU) per mL; the densities were estimated by serially diluting the culture in 0.9% sterile saline solution and direct plating the solute on tryptic soy agar with 5% sheep blood (Remel^TM^, Lenexa, KS, USA). Seven enrofloxacin concentrations were used for each isolate, normalized to 0 (control), 0.5, 0.75, 1, 2, 3, 5, and 10 multiples of the isolate’s MIC. The cultures with the antimicrobial were incubated aerobically with shaking (200 rpm) at 37 °C and sampled at 0, 1, 2, 3, 4, 5, 6, 7, 8, 12, and 24 hour of the incubation. The bacterial densities at each time point were estimated, similarly to as described above.

### Fitting PD model: model form

For each bacterial isolate, antimicrobial concentration, and time-kill experiment, the bacterial population density was plotted *vs.* time on a semi-logarithmic scale (the density log_10_-transformed). The population growth/decline rate was determined as the slope of the population density line during the initial linear phase. These rates together with the associated drug concentrations constituted the dataset. The relationships between the antimicrobial concentration and the bacterial population growth/decline rate were investigated by fitting to the dataset different forms of the *E*_max_ model. We investigated fit of a model formulated with an additive or fractional inhibitory PD-effect on the growth, and with or without a sigmoid form for the relationships. The models were fitted to the dataset as nonlinear mixed-effect regression models using the maximum likelihood method in the Phoenix^®^ NLME software (Certara USA, Inc., Princeton, NJ, USA). The fixed effect in the models was the antimicrobial concentration; the experiment was added as a random effect. Selection of the model form was based on the minimization of the Akaike Information Criterion (AIC); visual inspection of the goodness of fit of the predicted to the observed rate of the bacterial growth/decline at different drug concentrations; and visual inspection of the diagnostic plots for the residuals for outliers or trends which would suggest a poor model fit. Precision of the parameter estimates was also considered, and considered acceptable if the coefficient of variation was ≤25%.

### Fitting PD model: model’s parameter values

Once the best-fit PD model form was determined, the parameters’ values were compared among the three isolates. The values of a parameter were considered statistically significantly different between each two isolates if there was no overlap of the 95% Confidence Intervals (CI) for the parameter estimates.

### Simulating distributions of the time-kill curves

To visualize the antimicrobial PD against the bacterial isolates with different MIC values for the antimicrobial, we simulated the possible distributions of the rate of bacterial population growth/decline and the resulting bacterial density after a 1 hour exposure to different enrofloxacin concentrations. For each isolate, 1,000 Monte Carlo simulations of the best-fit PD model were performed with the parameters’ values obtained by Latin hypercube sampling^19^, from the parameters’ distributions assumed to each follow a Uniform (as in refs 20 and 21). The limits of the sampled distribution corresponded to the 95% CI of the parameter estimate, for each *E*_max_, *EC*_50_, or Hill coefficient, for that isolate (Table 1). The same distribution of the underlying bacterial population growth rate, *E*_0_, was sampled for all three isolates, a Uniform (0.24; 0.38), corresponding to the variability observed in the experiments (Table 1). Examination of the Cholesky decomposition of the matrix of variances and covariances from the non-linear mixed-effect regression model fit to the experimental data did not reveal any covariances among the parameters; therefore each parameter was sampled independently for the simulations. The simulations were implemented using the 4th order Runge-Kutta algorithm in Vensim^®^ Professional software (Ventana Systems Inc., Harvard, MA, USA). The outputs were plotted in SigmaPlot™ (Systat Software Inc., San Jose, CA, USA).

## Additional Information

**How to cite this article**: Wen, X. *et al*. Limitations of MIC as sole metric of pharmacodynamic response across the range of antimicrobial susceptibilities within a single bacterial species. *Sci. Rep.* **6**, 37907; doi: 10.1038/srep37907 (2016).

**Publisher's note:** Springer Nature remains neutral with regard to jurisdictional claims in published maps and institutional affiliations.

## Figures and Tables

**Table 1:** Estimates of pharmacodynamic parameters of enrofloxacin against *Pasteurella multocida* isolates with different MIC.

Isolate source	MIC(μg/mL)	Parameter	Estimate	CV (%)	2.5% CI of the estimate	97.5% CI of the estimate
Bovine lung	0.01	*E*_max_	1.64	8.47	1.34	1.93
		*EC*_50_, μg/mL	0.11	11.49	0.08	0.13
		*E*_0_, log(CFU/mL) x hour^−1^	0.32	8.86	0.26	0.38
		*H*	0.97	11.97	0.73	1.22
Bovine nasal swab	1.50	*E*_max_	0.81	9.07	0.65	0.96
		*EC*_50_, μg/mL	2.13	13.13	1.53	2.72
		*E*_0_, log(CFU/mL) x hour^−1^	0.31	10.40	0.24	0.38
		*H*	3.05	14.22	2.13	3.97
Bovine nasal swab	2.00	*E*_max_	0.69	4.37	0.63	0.75
		*EC*_50_, μg/mL	1.60	3.38	1.49	1.72
		*E*_0_, log(CFU/mL) x hour^−1^	0.29	3.11	0.27	0.30
		*H*	4.37	17.28	2.77	5.97
